# Global Brain Transcriptome Analysis of a *Tpp1* Neuronal Ceroid Lipofuscinoses Mouse Model

**DOI:** 10.1177/1759091419843393

**Published:** 2019-04-19

**Authors:** Miriam S. Domowicz, Wen-Ching Chan, Patricia Claudio-Vázquez, Judith G. Henry, Christopher B. Ware, Jorge Andrade, Glyn Dawson, Nancy B. Schwartz

**Affiliations:** 1 Department of Pediatrics, Biological Sciences Division, The University of Chicago, IL, USA; 2 Center for Research Informatics, Biological Sciences Division, The University of Chicago, IL, USA; 3 Department of Biochemistry and Molecular Biology, Biological Sciences Division, The University of Chicago, IL, USA

**Keywords:** neuronal ceroid lipofuscinoses, transcriptome, lysosomal tripeptidyl peptidase 1, pediatric neurodegeneration, neuroinflammation, circadian rhythm

## Abstract

In humans, homozygous mutations in the *TPP1* gene results in loss of tripeptidyl peptidase 1 (TPP1) enzymatic activity, leading to late infantile neuronal ceroid lipofuscinoses disease. Using a mouse model that targets the *Tpp1* gene and recapitulates the pathology and clinical features of the human disease, we analyzed end-stage (4 months) transcriptional changes associated with lack of TPP1 activity. Using RNA sequencing technology, *Tpp1* expression changes in the forebrain/midbrain and cerebellum of 4-month-old homozygotes were compared with strain-related controls. Transcriptional changes were found in 510 and 1,550 gene transcripts in forebrain/midbrain and cerebellum, respectively, from *Tpp1*-deficient brain tissues when compared with age-matched controls. Analysis of the differentially expressed genes using the Ingenuity™ pathway software, revealed increased neuroinflammation activity in microglia and astrocytes that could lead to neuronal dysfunction, particularly in the cerebellum. We also observed upregulation in the production of nitric oxide and reactive oxygen species; activation of leukocyte extravasation signals and complement pathways; and downregulation of major transcription factors involved in control of circadian rhythm. Several of these expression changes were confirmed by independent quantitative polymerase chain reaction and histological analysis by mRNA *in situ* hybridization, which allowed for an in-depth anatomical analysis of the pathology and provided independent confirmation of at least two of the major networks affected in this model. The identification of differentially expressed genes has revealed new lines of investigation for this complex disorder that may lead to novel therapeutic targets.

## Introduction

Neuronal ceroid lipofuscinoses (NCLs) are among the most common neurodegenerative diseases affecting the pediatric population, with an incidence estimated at 2 to 4 per 100,000 live births (Kollmann et al., 2013; Mink et al., 2013). Different forms of the disease were originally classified on the basis of the time of onset: infantile NCLs, late infantile NCLs, juvenile NCLs (JNCLs), and adult NCLs (Mink et al., 2013). Shared pathologies of the NCLs include progressive visual deterioration resulting in blindness, intellectual and motor decline, epilepsy, ataxia, spasticity, and premature death (Anderson et al., 2013; Schulz et al., 2013; Radke et al., 2015). The ultrastructural appearance of autofluorescent storage material found in patient tissues distinguishes different types of NCLs and has long been used as a diagnostic tool (Palmer et al., 2002, 2013). In the past 20 years, mutations in over a dozen genes have been characterized in families diagnosed with NCLs, and all but one type (Kufs disease) are inherited in an autosomal recessive manner (Carcel-Trullols et al., 2015; Mole and Cotman, 2015). NCLs are considered lysosomal storage diseases because most of the affected proteins have an endo/lysosomal network localization. However, some of these proteins are localized in other cellular compartments such as Golgi, endoplasmic reticulum, cytosol, plasma membrane, and even in the extracellular matrix (Carcel-Trullols et al., 2015) making it difficult to explain how all these gene defects lead to similar clinical symptoms and neurodegeneration. Furthermore, several of the affected genes are lysosomal hydrolases or peptidases, but their cellular substrates are incompletely elucidated, and the primary functions of the membrane proteins mutated in NCLs remain uncertain. Most of the advances in understanding the molecular pathology of NCLs have come from studies on patients’ fibroblast and lymphoblast cell lines and, increasingly, genetic animal models (dogs, sheep, mice, etc.; Cooper et al., 2006; Palmer et al., 2013; Cooper et al., 2015). Several hypotheses have emerged regarding potential cellular triggers for the disease: Evidence for cellular changes, including lysosomal dysregulation, endoplasmic reticulum stress, mitochondrial dysfunction, and compromised autophagy to name a few, have all been implicated (Palmer et al., 2013; Marotta et al., 2017). However, so far, none of these integrate all the genetic origins to a common biological pathway (Cooper et al., 2006; Palmer et al., 2013; Cooper et al., 2015; Marotta et al., 2017). Thus, it is conceivable that the various gene mutations implicated could alter different cellular processes that ultimately trigger a common chronic response that in turn is responsible for the neurodegenerative outcome.

From studies of some of the animal models, it is clear that astrogliosis and microglia activation play major roles in the disease (Pontikis et al., 2004; Pontikis et al., 2005; Parviainen et al., 2017) and precede neuronal cell death, such that neuronal loss is prevalent throughout the brain and at the end stages of the disease. There are specific brain areas of vulnerability at early stages such as the thalamocortical system and cerebellum (Cb;Cooper et al., 2006; Palmer et al., 2013;Cooper et al., 2015). Thus, astrogliosis and microglia activation are better predictors than the autofluorescent storage material in indicating initial areas of neuronal loss in the brains of many CLN models (Haltia et al., 2001; Tyynela et al., 2004; Anderson et al., 2013). Interestingly, no direct correlation has been found between the pattern of neuronal loss and the accumulation of storage material, and neither is the degree of neurodegeneration correlated with the amount of intracellular autofluorescent storage bodies accumulated (Cooper et al., 2006; Palmer et al., 2013; Cooper et al., 2015). Furthermore, it has been suggested that the accumulation of storage bodies can be neuroprotective because the few surviving neurons in the end stage of the disease are vastly distended with storage material (Tyynela et al., 2004; Cooper et al., 2015). Although NCLs have a strong neuronal toxicity component, the function of astroglia and microglia in disease progression is still unclear. Recently, stimulation of CLN3 deficient microglia and astrocytes in culture was shown to attenuate their ability to transform morphologically and alter their protein-secretion profiles (Parviainen et al., 2017). Also, there is evidence of aberrant astrocyte CX43 hemichannel activity in JNCL (Burkovetskaya et al., 2014).

We focused on the classical late infantile form of NCL (cLINCL), which in humans is caused by mutations in the *TPP1* gene (Sleat et al., 1997). The gene is also known as *CLN2* (ceroid lipofuscinosis neuronal 2), *GIG1*, *LPIC*, and *SCAR7* (NCBI Gene database, www.ncbi.nlm.nih.gov/gene). This disease variant is usually diagnosed between 2 and 4 years of age with onset of seizures followed by the severe neurological deterioration common to all NCLs, and most patients die between 7 and 15 years of age (Sleat et al., 1997; Fietz et al., 2016). The only currently available treatment is a recent FDA-approved enzyme replacement therapy in which a recombinant form of human TPP1 (Brineura® [cerliponase alfa]) is infused into the cerebral spinal fluid of patients. This treatment appears to reduce the loss of walking ability in human trials (clinical trial NCT01907087), but it is expensive and may lead to many side effects due to the prolonged nature of the treatment (Schulz et al., 2018).

A well-established mouse model to study cLINCL was generated and characterized by targeting the mouse gene *Tpp1* in the Lobel laboratory (Sleat et al., 2004), resulting in loss of detectable TPP1 activity, progressive neurological phenotypes including ataxia, and increased motor deficiency. Affected mice have a median survival of 19 weeks with motor deficiencies detectable after 10 weeks, thus representing an excellent model of the human disease progression. More recently, Pierce’s laboratory independently made a *Tpp1* knockout model with a more severe phenotype (Geraets et al., 2017), but the Lobel model has been better characterized, including its ability to respond to protein replacement therapies (Passini et al., 2006; Chang et al., 2008).

Our laboratory has extensive experience in microglia and astrocyte responses to embryonic and perinatal brain injury (Domowicz et al., 2011; Domowicz et al., 2018). Because these cell types play fundamental roles in neuronal survival and injury repair and express *Tpp1* at higher levels in astrocytes (7 times more) and microglia (4 times more; Zhang et al., 2014) than in neurons, they could be major contributors to the pathology of cLINCL. The objective of this study was to perform, for the first time in a cLINCL model, a transcriptome-wide characterization by RNA sequencing (RNA-seq) of two macroareas mostly affected in the 4-month-old *Tpp1^–^*^/^*^–^* (Cb and forebrain/midbrain [F/M]). Because we were particularly interested in the microglia and astrocyte response and both cell types have been known to quickly alter their transcriptome profiles in culture (Haimon et al., 2018), we used whole Cb to perform these analyses. We then matched the differentially expressed genes (DEG) in the *Tpp1* model to the already reported individual cell transcriptomes (Zhang et al., 2014; Lun et al., 2015; Gosselin et al., 2017; Boisvert et al., 2018; Haimon et al., 2018; Olah et al., 2018). Our results in 4-month-old *Tpp1^–^*^/^*^–^* mouse F/M and Cb show strong neurodegenerative inflammatory responses involving microglia and astrocytes, activation of leukocyte extravasation, dysregulation of neurotransmitter production, and upregulation of nitric oxide (NO) and reactive oxygen species (ROS); in contrast, we observed downregulation of transcription factors involved in control of circadian rhythm. This study represents, to our knowledge, the first analysis of this kind made in any animal model for cLINCL.

## Materials and Methods

### Mice

All animal procedures were performed according to the University of Chicago Institutional Animal Care and Use Committee regulatory policies. Mice were housed under standard conditions, with access to food and water *ad libitum*, in a 12-hr light and dark cycle. The mouse model for cLINCL, in which *Tpp1* is disrupted by gene targeting, has been previously described (Sleat et al., 2004) and is referred in this article as the *Tpp1^–/–^* mutant. The mouse line was a gift from Drs. P. Lobel and D. E. Sleat. The line is being maintained by *Tpp1^+/–^* heterozygous crosses. In all experiments, *Tpp1^–^*^/^*^–^* homozygous mice were compared with heterozygous (*Tpp1^+/–^*) or wild-type (*Tpp1^+/+^*) siblings of matched age and sex, as indicated.

### Gene Expression Analysis

Total RNA was obtained from the Cb and F/M of three 4-month-old *Tpp1^–^*^/^*^–^* and wild-type mice by TRIzol (Thermo Fisher Scientific, Waltham, Massachusetts, USA) extraction followed by a QIAGEN RNeasy Kit® cleanup procedure (QIAGEN, Hilden, Germany) which included a DNase digestion step. RNA quality (RIN > 9.5) assessments, cDNA library preparation, and single-end sequencing with 50-nucleotide reads were performed by the University of Chicago Genomics Facility on Illumina platforms. RNA sequence files were transferred to the University of Chicago Center for Research Informatics’ Tarbell High-Performance Computing cluster for analysis.

The quality of raw sequencing data was assessed using FastQC v0.11.5 (Andrews, 2010); Illumina adapter/primer sequences were detected from sequencing reads. All RNA reads were first mapped to the mouse (GRCm38) reference genome using STAR v2.5.2b release with default parameters (Dobin et al., 2013). Picard v2.8.1 (http://broadinstitute.github.io/picard/) was used to collect mapping metrics.

The resulting files from the previous alignment step in the RNA-seq analysis were taken individually as input to quantify transcriptional expression using Cufflinks v2.2.1 (Trapnell et al., 2010; RRID:SCR_014597) and Rsubread:featureCounts v1.26.1 (Liao et al., 2014; RRID:SCR_009803). Afterward, several methods of differential expression analysis (Costa-Silva et al., 2017), including Cuffdiff v2.2.1 (Trapnell et al., 2010; RRID:SCR_014597), edgeR v3.18.1 (Robinson et al., 2010; RRID:SCR_012802), DESeq2 v1.16.1 (Love et al., 2014; RRID:SCR_015687), and limma v3.32.10 (Ritchie et al., 2015; RRID:SCR_010943), were used to discover DE-mRNA genes (fold change ≥ 1.5 and false discovery rate [FDR]) < 0.1 between pairwise groups based on the expression estimate of individual gene mRNAs. To obtain the groups with similar expression trends based on the identified DE-mRNA genes, several in-house scripts were implemented using R (https://www.rproject.org/) and Python (https://www.python.org/) languages.

The identified DE genes were further used as input to functional analysis modules for the identification of enrichment of functional categories and regulatory networks, using Gene Ontology (GO) terms (RRID:SCR_002811) and KEGG-enrichment analyses (RRID:SCR_012773), as well as Ingenuity Pathway Analysis (IPA; RRID:SCR_008653). Pathways significantly enriched in the genes of interest were identified using clusterProfiler (Yu et al., 2012; v3.6.0) at FDR-adjusted *p* value < .10 (hypergeometric test). Gene Set Enrichment Analysis was performed using clusterProfiler (Yu et al., 2012; v3.6.0; RRID:SCR_016884) as well.

The cell-type specificities of the DEG were determined based on transcriptome data from Zhang et al. (2014; http://web.stanford.edu/group/barres_lab/brain_rnaseq.html). Briefly, genes expressed in excess of fivefold higher in each cell type (neurons, astrocytes, microglia, oligodendrocytes, and endothelial cells) with respect to the bulk expression of all other cell types were considered enriched in that cell type. The individual datasets for each cell type were then matched to the DEG in each brain sample to assign cell-type specificity.

### mRNA *In Situ* Hybridization

Control and *Tpp1* mutant 4-month-old brains were fixed in 4% paraformaldehyde and processed for nonradioactive *in situ* hybridization (ISH) as described previously (Domowicz et al., 2008, 2011). To prepare the digoxigenin (DIG)-labeled RNA probes used for ISH, cDNA fragments from the glial fibrillary acidic protein (*Gfap*), period1 (*Per1*), and CD68 antigen (*Cd68*) genes were generated by PCR and inserted into pCRII dual-promoter vector plasmids (Invitrogen, Waltham, MA, USA). Sequencing of the cloned gene fragments was performed with an ABI PRISM 377XL sequencer (Perkin Elmer, Waltham, MA, USA) by the University of Chicago Cancer Center DNA sequencing facility. Riboprobes incorporating DIG-labeled nucleotides were synthesized from linearized PCR templates with SP6 or T7 RNA polymerase (Roche, Indianapolis, IN, USA). Probed mRNAs were detected after hybridization with an alkaline phosphatase-conjugated anti-DIG antibody (Roche, Indianapolis, IN, USA). Alkaline phosphatase activity was detected (blue color) using BCIP and NBT substrates (Roche, Indianapolis, IN, USA). A virtual library of the images was obtained using a CRi Pannoramic Scan Whole Slide Scanner (Perkin Elmer, Waltham, MA, USA), and final pictures were selected using the Pannoramic Viewer Software (RRID:SCR_014424).

Quantification of the number of CD68+ cells and sizes in *Tpp1* mutant and control brains were performed on 10 cortical and thalamic fields using the Image J software package (RRID:SCR_003070). Unfortunately, levels of Cd68 mRNA in wild-type brain were too low to make a reliable quantification.

### Quantitative Real-Time Reverse Transcription Polymerase Chain Reaction

RNA purified as described earlier was reverse transcribed with the High-Capacity Reverse Transcription Kit (Applied Biosystems, Waltham, MA, USA), and quantitative real-time reverse transcription polymerase chain reactions (RT-qPCRs) were performed using the SsoAdvanced™ Universal SYBR Green system (Bio-Rad Laboratories, Hercules, CA, USA) in a CFX-96 real-time instrument (Bio-Rad Laboratories, Hercules, CA, USA). For each gene, forward and reverse primers were designed using Primer-BLAST (NCBI; RRID:SCR_003095) and later tested in RT-qPCR; only primer pairs with 90% to 105% efficiency were used. Relative normalized expression values were determined using the ΔΔCt methods for the indicated target genes relative to *Gapdh* (glyceraldehyde-3-phosphate dehydrogenase) in the wild-type mouse sample. The data are expressed as means ± standard deviations. Determinations were performed in triplicate, and experiments were repeated with independent RNA samples at least three times with consistent results. Statistical significances were evaluated by the paired-samples Student’s *t* test. Values of *p* < .05 for the null hypothesis were considered significant.

## Results

### *Tpp1*-Mutant Brain Transcriptome Analysis

**Figure 1.**
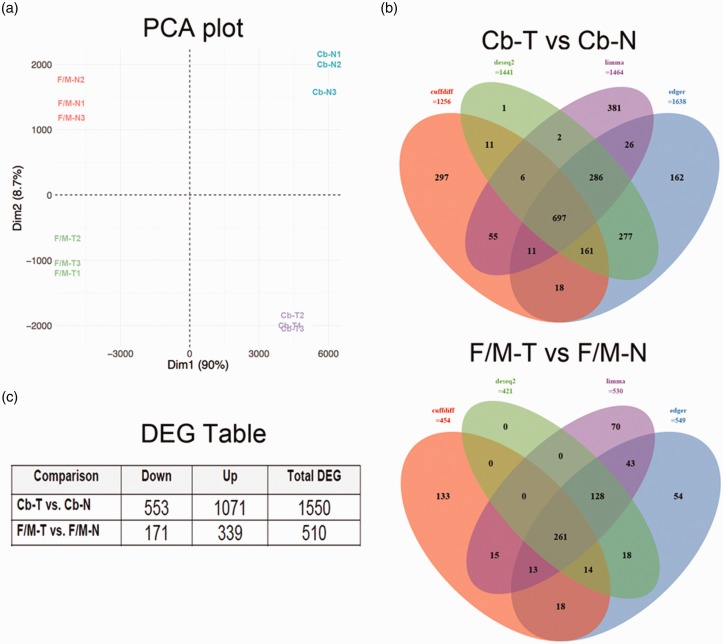
Statistical determination of DEG. (a) PCA plot of all samples based on normalized expressions. Control forebrain/midbrain (F/M-N), *Tpp1^–^*^/^*^–^* forebrain/midbrain (F/M-T), control cerebellum (Cb-N), and *Tpp1^–^*^/^*^–^* cerebellum (Cb-T) of triplicate samples are represented. (b) Venn diagram of differentially expressed mRNA genes identified by four different methods: Cuffdiff, edgeR, DESeq2, and limma. Only genes identified by at least two methods were used for further analysis. (c) Number of up-/downregulated DEG in different comparison(s). PCA = principal component analysis; DEG = differentially expressed genes.

To analyze the pattern of DEG underlying the pathology in this end-stage model of cLINCL, RNA-seq of F/M and Cb RNA from 4-month-old *Tpp1^–^*^/^*^–^* (T) and wild-type control (N) brains was conducted. Readouts obtained from RNA-seq (and passing quality control) were aligned to the UCSC *Mus musculus* mm10 reference genome; 88.3% of the reads were successfully mapped to the mouse reference genome. After eliminating low-expressed-mRNA genes, 15,294 genes out of 52,550 annotated mouse genes were further analyzed. Dimensionality reduction, an informative approach for clustering and exploring the relationships between samples, was conducted with the principal component analysis plot based on the normalized mRNA expression profiles. As presented in Figure 1(a), four obvious groups were separated in terms of tissues and treatments. To identify DE-mRNA genes between pairwise groups, several state-of-the-art tools (Costa-Silva et al., 2017), including Cuffdiff, DEseq2, edgeR, and limma were used. Two pairwise comparisons, F/M-T versus F/M-N and Cb-T versus Cb-N, were conducted using the criteria of fold changes greater than 1.5 and FDR less than 0.1. To identify a more robust collection of intrinsically regulated genes, the overlapped DEG detected by more than one method were considered as the final set of DEG for further analysis. Venn diagrams of overlapping DEG identified from the previously mentioned methods are shown in Figure 1(b). A total of 1,550 and 510 mRNAs were found to be differentially expressed in Cb and F/M areas, respectively (Figure 1(c)).

**Figure 2.**
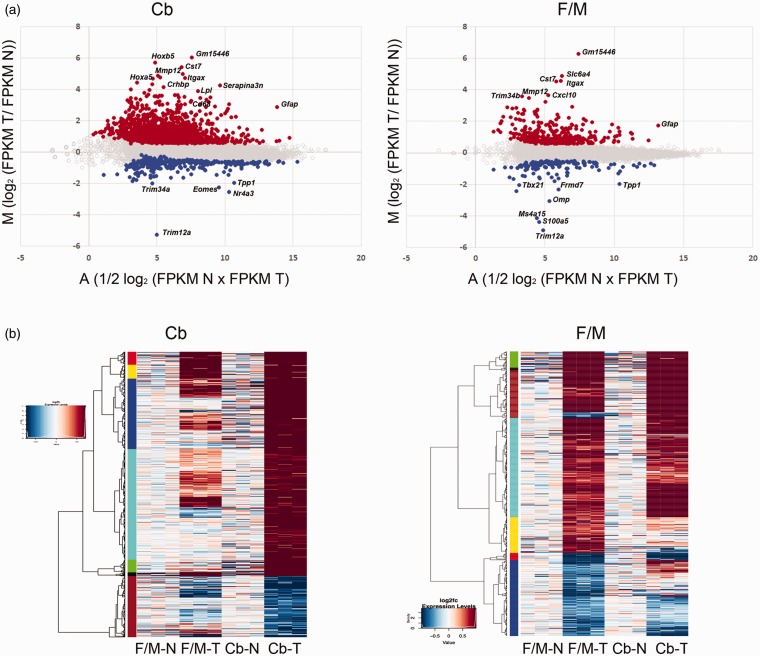
Visualization of DEG. (a) MA plot representing the ratio of FPKM expression values between *Tpp1^–^*^/^*^–^* cerebellum (Cb) and forebrain/midbrain (F/M) areas and their corresponding control areas (N) plotted against their average. The statistically significant genes with >1.5 fold change and false discovery rate of less than 0.1 are plotted in orange color (upregulated genes) and blue (downregulated genes). The rest of the genes are represented with gray open circles. (b) Heat map displaying the 1,550 and 510 genes that are differentially expressed between Wt (N) versus *Tpp1^–^*^/^*^–^* (T) cerebellum (Cb) and forebrain/midbrain (F/M), respectively. Representation of the same genes in complementary samples is also included for comparison. Each column represents an individual triplicate. Red color represents relative increase in abundance, blue color represents relative decrease, and white color represents no change as measured by log2fc (Log_2_ of T vs. N ratio) and represented in the inset. The rows are organized by hierarchical clustering (represented at the left). FPKM= Fragments Per Kilobase of transcript per Million mapped reads.

The differential gene expressions in the *Tpp1^–^*^/^*^–^* samples compared with controls are visualized in Figure 2, where the MA plots present the ratio of FPKM (Fragments Per Kilobase of transcript per Million mapped reads) expression values between the two conditions. All 15,294 genes are represented in the plot with DEG highlighted in different colors (Figure 2(a)). The heatmap in Figure 2(b) demonstrates the hierarchical clustering between the three controls and *Tpp1^–^*^/^*^–^* samples from Cb and F/M areas, thus illustrating differential expression of the significantly regulated genes. For comparison, the levels of expression of the Cb and F/M areas are plotted in both heatmaps. It is noteworthy that a considerable number (299) of the genes whose expressions are significantly changed in Cb also have changed expression (or have a tendency to change; Table S1) in the same direction in the F/M area and *vice versa*. This finding suggests that many of the networks and pathways implicated in the disease are likely affected in the whole brain but that the Cb is perhaps further along (more affected) in developing the pathology. A full list of DEG is provided in supplemental data (Table S1), and all sequence FASTQ files are available from the Gene Expression Omnibus (accession number # GSE123509).

### DEG Cell Type Profile Analysis

**Figure 3.**
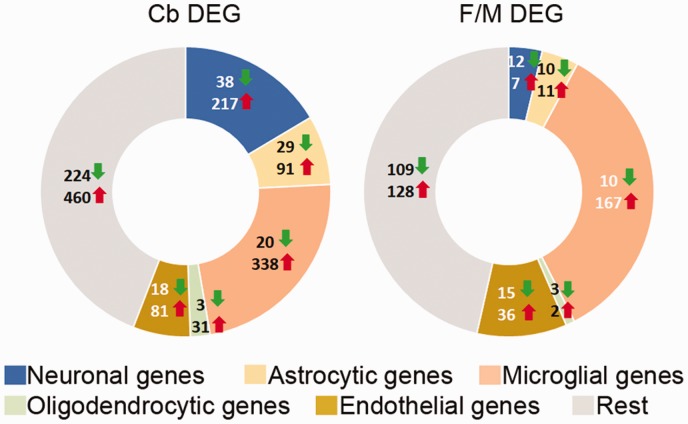
Identification of cell-type-specific DEG in wt and *Tpp1^–^*^/^*^–^* brain regions. Distribution of cell-type-specific genes that are commonly enriched (upregulated: red up arrow; downregulated green down arrow) in between DEG from Cb-T versus Cb-N and F/M-T versus F/M-N comparisons. Cell-type specificity of DEG was established from data imputed from Zhang et al. (2014; http://web.stanford.edu/group/barres_lab/brain_rnaseq.html) based on genes that were exclusively expressed in each cell type and had FPKM expression values at least five time higher than in the rest of the other cell types. DEG = differentially expressed genes.

We also determined the cell-type enrichment profile for both sets of DEG based on reported cell-type transcriptomes (Zhang et al., 2014; Figure 3). Even though 44% to 46% of the genes are unassigned (probably because they are expressed in several cell types), these data indicate that the microglial transcriptome is highly altered in the *Tpp1^–^*^/^*^–^* animals, followed in descending order of alteration by those of neurons, astrocytes, endothelial cells, and the least-affected cell types, oligodendrocytes. The most strongly upregulated genes in astrocytes from both F/M and Cb were *Serpina3n*, *Gfap*, *C4b*, and *Aspg*, which were all found to be expressed in reactive astrocytes after lipopolysaccharide injection or middle cerebral artery occlution brain injury (Zamanian et al., 2012) and are also elevated in aging astrocytes (Clarke et al., 2018). Many genes were associated with activated microglia; among the largest changes are *Itgax*, *Ccl3*, *C3*, and *Rgs1*, which have also been shown to be increased in human aging microglia (Olah et al., 2018).

**Figure 4.**
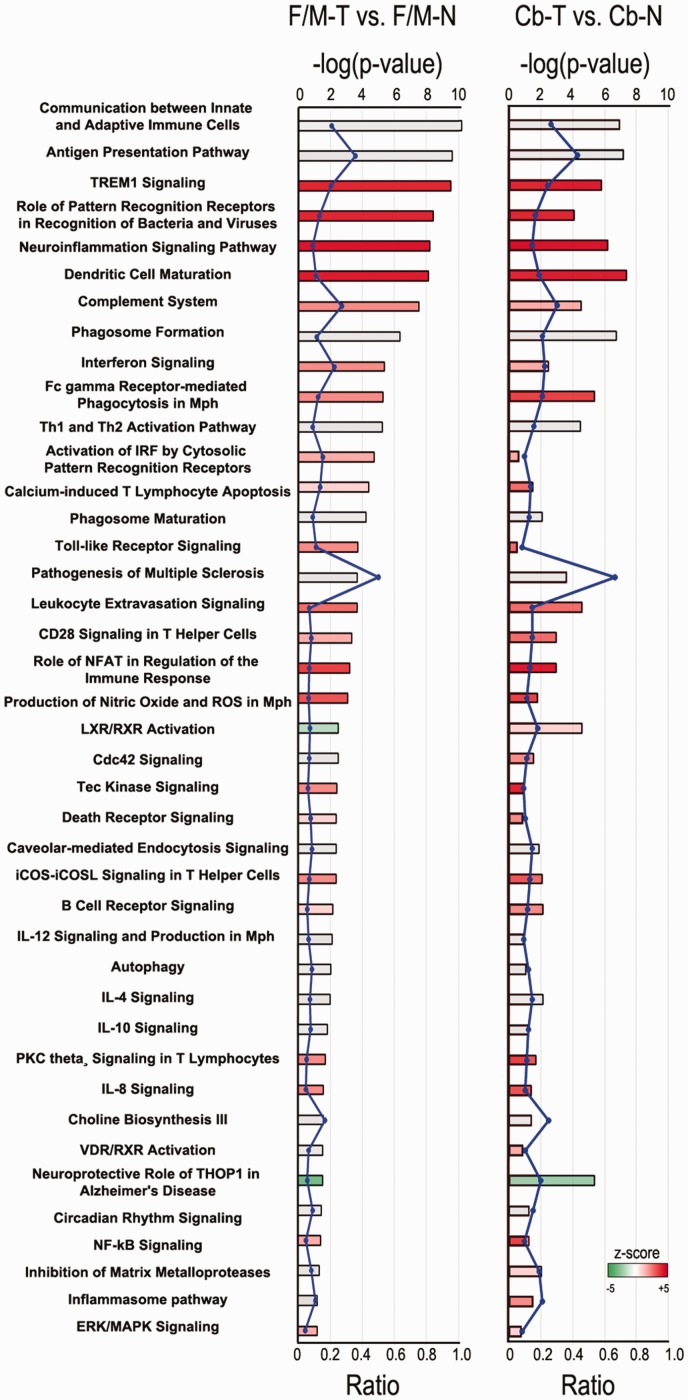
Ingenuity Pathway Analysis (IPA) from DEG in *Tpp1^–^*^/^*^–^* versus N mouse brain. Selected canonical pathways identified using IPA gene ontology algorithms for F/M and Cb areas scored as −log(*p* value) from Fisher’s exact test, set here to a threshold of 1.25. Bars are colored according to the *z* score (positive *z* score is red, negative *z* score is green, and no activity pattern available is gray). The ratio (blue dots connected by a line) indicates the ratio of genes from the dataset that map to the pathway divided by the total number of genes that map to the same pathway. For a complete list of canonical pathways and genes involved, see Table S2 and S3. F/M-N = control forebrain/midbrain; F/M-T = *Tpp1^–^*^/^*^–^* forebrain/midbrain; Cb-N = control cerebellum; Cb-T = *Tpp1^–^*^/^*^–^* cerebellum; NFAT = nuclear factor of activated T-cells; ROS = reactive oxygen species; LXR = liver X receptor; RXR = retinoid X receptor; iCOS = inducible T-cell costimulator; iCOSL = inducible T-cell costimulator ligand; IL = interleukin; VDR = vitamin D receptor; PKC= protein kinase C; NFκB = nuclear factor kappa-light-chain-enhancer of activated B cells; ERK = extracellular signal-regulated kinases; MAPK = mitogen-activated protein kinases.

In addition, cellular processes and upstream regulators associated with the DEG found in our *Tpp1^–^*^/^*^–^* samples relative to controls were identified using QIAGEN’s IPA® (www.qiagen.com/ingenuity). A selected list of canonical pathways detected are represented in Figure 4 including a representation of the modified gene expression ratio (number of genes from the DEG list that map to the pathway divided by the total number of genes mapping to the same pathway) and a representation of the *z* score, where a positive *z* score (red) indicates a predicted activation, and a negative *z* score (green) indicates a predicted inactivation of the enriched pathway. Several of these canonical pathways, which can be considered relevant to this disease, are further highlighted in Table 1, with a detailed list of DEG found to be involved in each pathway. For a complete list of canonical pathways and involved DEG, see Table S2 for Cb and Table S3 for F/M areas.

**Table 1.** Selected Canonical Pathways and Associated Differentially Expressed Genes in Tpp1*^–^*^/^*^–^* Versus Wild-Type Cerebellum (Cb) and Forebrain/Midbrain (F/M).

Tissue	Ingenuity canonical pathways	–log(*p* value)	Ratio	*z* score	DEG
Cb	TREM1 signaling	5.82	0.246	3.638	Icam1, Tyrobp, Nlrp10, Lat2, Itga5, Cd83, Fcgr2b, Tlr9, Tlr2, Nlrc5, Ccl2, Plcg2,Tlr7, Tlr13, Cd86, Tlr3, Itgax
F/M		9.52	0.203	3.207	Sigirr, Nlrp3, Icam1, Tyrobp, Lat2, Fcgr2b, Tlr2, Nlrc5, Ccl12, Tlr1, Tlr7, Tlr13, Cd86, Itgax
Cb	Neuroinflammation signaling pathway	6.2	0.147	5	B2m, Gabra5, Tgfbr1, Icam1, Bdnf, Pycard, H2-Ab1, H2-Aa, Cx3cr1, Ccl5, H2-Q6, Cxcl10, Gabrg3, Hmox1, Ccl12, H2-DMa, Tgfb1, Pik3cg, Pla2g5, Cybb, Tlr7, Tlr3, Gabrq, Tnfrsf1a, Tyrobp, Pla2g4c, Pla2g3, Tlr9, Csf1r, Tlr2, Slc6a11, Irf7, Gabrr2, Trem2, Syk, Plcg2, Pla2g4b, Ncf2, Cd86, Tlr13, Mmp9, H2-Eb1
F/M		8.21	0.0877	4.491	B2m, H2-Oa, Nlrp3,Icam1,Ager,Tyrobp,H2-Q6,H2-Ab1, Ccl5, Ngf, Tlr2, Cxcl10,Hmox1, Irf7,Ccl12,Tgfb1, Trem2, Ncf2,Tlr1, Cybb, Tlr7, Tlr13, Cd86, Stat1, H2-Eb1
Cb	Complement system	4.5	0.303	1.633	C4a/C4b, Itgb2, Itgam, C3, Masp2, C1qc, C1qa, C1qb, C3ar1, Itgax
F/M		7.52	0.273	2.236	C4a/C4b,Itgb2,Cd59,C3,C1qc,C1qa,C1qb, C3ar1, Itgax
Cb	Role of NFAT in regulation of the immune response	2.93	0.131	4.69	Blnk, Gng4, Plcb2,H2-Q6,Fcgr2a,H2-Aa, H2-Ab1, Fcgr2b,Tlr9, Fcgr1a, Btk, Lck, Gna15,H2-DMa, Pik3cg, Plcg2,Syk, Zap70, Fcer1g,Cd86lcp2,H2-Eb1, Fcgr3a/Fcgr3b
F/M		3.22	0.0682	3.464	Btk, H2-Oa, Gna15, H2-Q6, Fcgr2a, H2-DAa, Fcer1g, Cd86, Fcgr2b, Fcgr1a, H2-Eb1, Fcgr3a/Fcgr3b
Cb	Leukocyte extravasation signaling	4.55	0.146	2.887	Rac2, Icam1, Rock2, Timp1, Pik3cg, Cyba, Cybb, Mmp12, Mmp19, Timp2, Pxn, Cxcr4, Itga5, Mmp2, Rapgef3, Ncf4, Tlr9, Selplg, Btk, Itgb2, Ncf1, Itgam, Arhgap9, Was, Plcg2, Ncf2, Cd44, Vav1,Cldn14, Mmp9
F/M		3.67	0.0683	2.309	Rac2, Icam1, Ncf4, Btk, Itgb2, Was, Timp1, Cyba, Prkcd, Ncf2, Cybb, Cldn14, Vav1, Mmp12
Cb	Phagosome formation	6.71	0.208	n.a.p.a	Mrc1, Plcb2, Fcgr2a, Itga5, Plcl2, Plch2, Fcgr2b, Tlr9, Fcgr1, Inpp5d, Tlr2, Itgb2, Fcrls, Rhov, Itgam, Syk, Pik3cg, Plcg2, Fcer1g, Tlr7, Tlr13, Tlr3, C3ar1, Fcgr3a/Fcgr3b, Itgax
F/M		6.33	0.117	n.a.p.a	Tlr2, Itgb2, Fcrls, Fcgr2a, Prkcd, Tlr1, Tlr7, Fcer1g, Tlr13, Fcgr2b, Fcgr1a, C3ar1, Fcgr3a/Fcgr3b, Itgax
Cb	Phagosome maturation	2.09	0.124	n.a.p.a	B2m, Lpo, H2-Q6, Ctsw, Dynlt1, Tap1, Prdx6, Ctsz, Ctsd, Tubb6, Ctsh, Ctss, Ncf2, Rab7b, Cybb, Ctsc, H2-Eb1
F/M		4.24	0.0876	n.a.p.a	B2m, Ctsz, Ctsd, Ctss, Ctsh, H2-Q6, Lpo, Ncf2, Cybb, Ctsc, Tap1, H2-Eb1
Cb	Production of nitric oxide and reactive oxygen species	1.77	0.109	3.771	Apoe, Ptpn6, Tnfrsf1a, Map3k13, Ncf4, Tlr9, Spi1, Irf1, Tlr2, Rhov, Lyz, Ncf1, Pik3cg, Plcg2, Cyba, Ncf2, Cybb, Irf8, Tnfrsf1b, Clu
F/M		3.07	0.0656	2.53	Tlr2, Lyz, Ptpn6, Prkcd, Ppm1j, Cyba, Ncf2, Cybb, Ncf4, Irf8, Stat1, Spi1
Cb	Toll-like receptor signaling	0.48	0.0833	2.449	Tlr2, Il33, Tlr7, Cd14, Tlr3, Tlr9
F/M		3.71	0.111	1.89	Sigirr, Tlr2, Ly96, Il1a, Tlr1, Tlr7, Cd14, Eif2ak2
Cb	Autophagy	1.05	0.119	n.a.p.a	Ctsz, Ctsd, Ctss, Ctsh, Ctsw, Ctsc, Atg9b
F/M		2	0.0847	n.a.p.a	Ctsz, Ctsd, Ctss, Ctsh, Ctsc
Cb	Circadian rhythm signaling	1.21	0.152	n.a.p.a	Per1, Vipr2, Adcyap1, Vip, Per2
F/M		1.44	0.0909	n.a.p.a	Per1, Vipr2, Per2
Cb	Inflammasome pathway	1.49	0.211	2	Naip, Aim2, Pycard, Panx1
F/M		1.18	0.105	n.a.p.a	Naip, Nlrp3

*Note*. DEG = differentially expressed genes; n.a.p.a. = no activity pattern available; NFAT = nuclear factor of activated T-cells.

### Neuroinflammation Signaling Pathway

**Figure 5.**
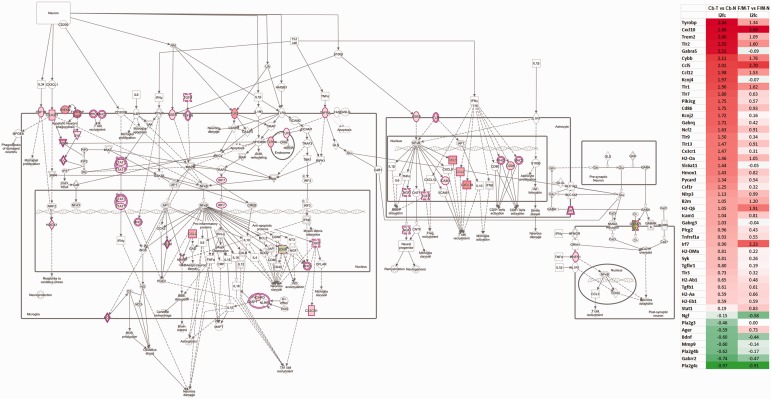
Neuroinflammation pathway. Canonical pathway analysis by IPA highlights the genes affected in the neuroinflammation pathways for Cb and F/M. Involved DEG detected by RNA-seq analysis and change ratio (l2fc=log_2_ of T. N ratio) are indicated to the right. F/M-N = control forebrain/midbrain; F/M-T = *Tpp1^–^*^/^*^–^* forebrain/midbrain; Cb-N = control cerebellum; Cb-T = *Tpp1^–^*^/^*^–^* cerebellum.

The list of DEG reveals a large number of genes with roles in the inflammatory process, in particular those reported to be expressed by microglia and astrocytes at their activated stage; thus, it is not surprising to see the neuroinflammation signaling pathway as one of those most significantly altered in the *Tpp1^–^*^/^*^–^* Cb and F/M in our IPA (Figures 4 and 5; Table 1). As indicated, 25 upregulated genes were found in the F/M area, and 42 altered expression genes were found in Cb (36 upregulated and 6 downregulated), all associated with the neuroinflammation pathway (Figure 5). In-depth analysis of the genes affected in this pathway highlights the possible regulatory process activated in microglia and astrocytes which may lead to altered postsynaptic behavior (Figure 5), in particular in Cb where gamma-aminobutyric acid (GABA) C receptor, subunit rho 2 (*Gabrr2*) is strongly downregulated. More than 75% of the DEG associated with this pathway in F/M areas were also altered in Cb, indicating similar inflammatory processes are occurring in both areas, but with a higher positive *z* score, that is, stronger involvement, in the Cb where more gene expressions are altered, and levels of gene activation are higher (Figure 5).

IPA analysis also identifies, in both brain areas, several highly significant upstream regulators; that is, the transcription factors, *Irf3* (F/M: *p* < 8.5E-36, *z* = 5.2; Cb: *p* < 6.6E-17, *z* = 4.7); *Irf7* (F/M: *p* < 1.1E-28, *z* = 4.6; Cb: *p* < 5.7E-15, *z* = 4.1); the interleukin *Ifng* (interferon gamma; F/M: *p* < 9.9E-33, *z* = 6.3; Cb: *p* < 1.5E-26, *z* = 7.1); and interferon alpha/beta receptors (*Ifnar1* and *Ifnar2*; F/M: *p* < 1.4E-44, *z* = 6.2; Cb: *p* < 1.5E-25, *z* = 6.2). Among the factors in this list, only Irf7 message is actually upregulated in both brain areas. Furthermore, Irf7, a critical transcription factor for the induction and positive feedback regulation of type I IFN signaling (Seth et al., 2006), has been found upregulated in experimental autoimmune encephalomyelitis (EAE; Salem et al., 2011) and is known to play a critical role with Tgfβ in microglia inflammatory response (Cohen et al., 2014).

Many of the highly significantly modified canonical pathways are associated with the immune system response and are consistent with findings for other neurodegenerative diseases such as multiple sclerosis (MS) and Alzheimer’s disease (AD; see Figure 4). For example, the use of single-cell RNA-seq gene expression profiling recently allowed the characterization of Alzheimer’s disease-associated microglia (DAM; Deczkowska et al., 2018) as having increased levels of *Trem2* and *Tyrobp* expression, which was consistent with the increases observed in our data (Figure 5).

### Histological and Quantitative Changes Associated With Astrocytes and Microglia

**Figure 6.**
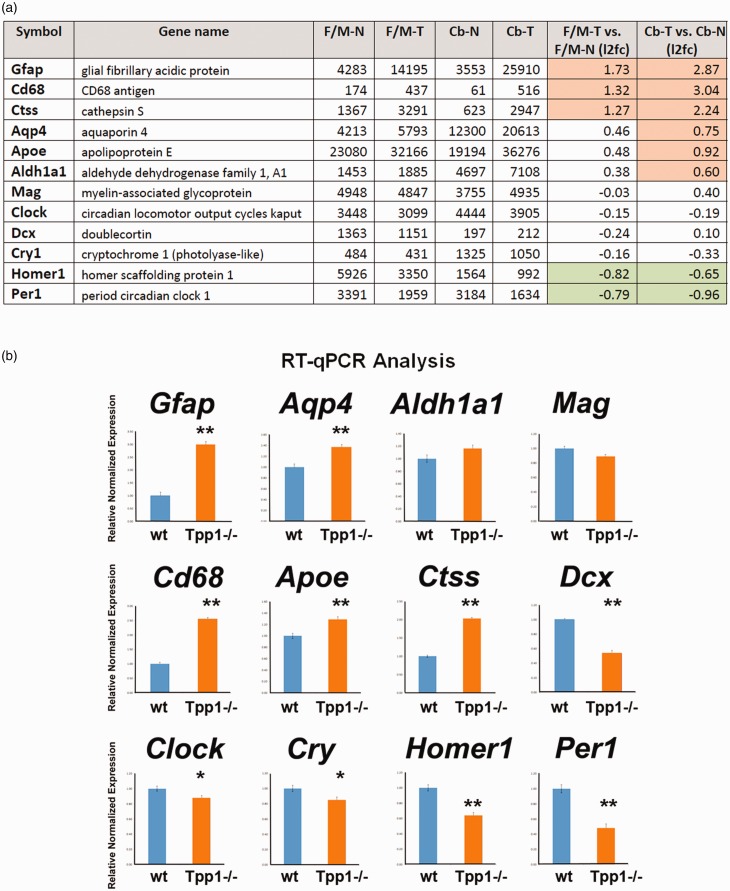
Corroboration of RNA-seq results by RT-qPCR for selected genes. (a) Table counts per million (CPM) data from RNA-seq study and log2 of *Tpp1^–/–^* (T) versus control (N) ratio (l2fc) in Cb and F/M for 12 genes. Significant change ratios are highlighted in pink (upregulated) and green (downregulated genes). (b) Relative transcript expression of the same 12 genes quantified by qPCR in independent F/M RNA extractions, performed in triplicate. Relative normalized expression values were determined from the ΔΔCt for the indicated target genes relative to Actb (β–actin) expression in control brains. *p < .02; **p < .0001. F/M-N = control forebrain/midbrain; F/M-T = *Tpp1^–^*^/^*^–^* forebrain/midbrain; Cb-N = control cerebellum; Cb-T = *Tpp1^–^*^/^*^–^* cerebellum; l2fc=log_2_ of T. N ratio; Tpp1 = tripeptidyl peptidase 1; RT-qPCR = quantitative real-time reverse transcription polymerase chain reaction; *Gfap* = glial fibrillary acidic protein; *Aqp4 =* aquaporin 4; *Cd68 =* CD68 antigen; *Per1 =* period1.

To confirm and validate the RNA-seq data, several genes were selected, and transcriptional differences were confirmed by RT-qPCR and mRNA ISH. The genes chosen encompass a broad range of expression levels and include significant (highlighted in color) and nonsignificant up- and downregulated genes (Figure 6(a)). Consistent results were obtained for Cb and F/M samples; thus, only F/M samples are represented (Figure 6(b)). The magnitude and directions of the differences obtained by qPCR are consistent with the RNA-seq data. Moreover, in F/M areas, tendencies in the RNA-seq that do not reach significance, such as *Apoe* and *Aqp4*, were confirmed to be significantly changed by qPCR (Figure 6(b)). These results may indicate that due to the high level of stringency in the statistical analysis of RNA-seq, the differences reported by this technique are underestimating the total numbers of differentially expressed transcripts and stress the need to extend the assessment of DEG to other techniques as well.

**Figure 7.**
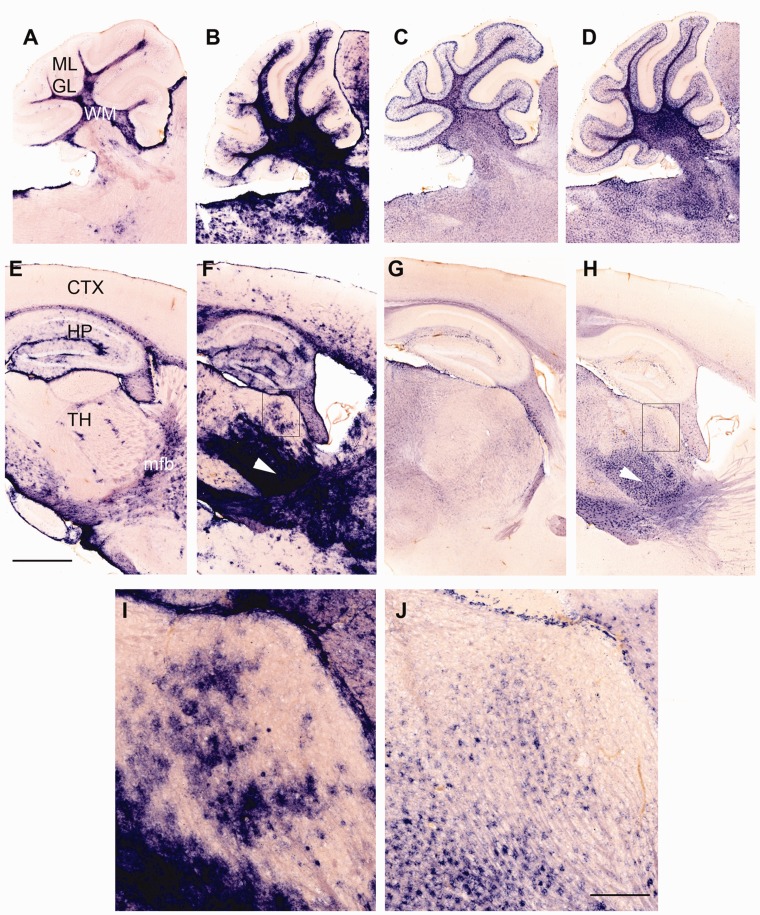
Altered expression patterns of astrocytic markers in *Tpp1^–^*^/^*^–^* 4-month-old brains. Expression patterns of GFAP (a, b, e, f, i) and Aqp4 (c, d, g, h, j) transcripts in *Tpp1^–^*^/^*^–^* (b, f, i and d, h, j) and control (a, e and d, h) in 4-month-old brains by *in situ* hybridization (blue staining). Cerebellar (a to d) and forebrain areas (e to i) are shown. (i) and (j) depict close-ups of areas indicated in (f) and (h). White arrowhead in (f) and (h) indicates ventral thalamus. Scale bars: (a) to (g) = 1,000 μm; (i) and (j) = 200 μm. ML = molecular layer; GL = granular layer; WM = molecular matter; CTX = cortex; HP = hippocampus; TH = thalamus; mfb = medial forebrain bundle.

Transcripts from astrocytes and microglia were found to be strongly dysregulated in the Tpp1*^–^*^/^*^–^* mouse brain on the basis of our DEG cell-type enrichment profile (Figure 3); thus, we analyzed the histopathological changes in these two cell types using mRNA ISH. For astrocytes, ISHs in brain sagittal sections were performed using *Gfap* and *Aqp4* probes (Figure 7). The *Gfap* transcript, which is well known to be upregulated in reactive astrocytes (Pekny et al., 2014), was found to be increased in all areas of the brain in a patchy or punctuated pattern (Figure 7(f) and (i)). Certain areas such as the ventral thalamus, medial forebrain bundle (Figure 7(f) and (i)), cerebellar white matter, and granular layer (Figure7(b)) were more distinctly affected, confirming the previously described distribution of reactive astrocytes in *Tpp1^–/–^* brains by immunohistochemistry (Sleat et al., 2004). The aquaporin 4 (*Aqp4*) transcript, which has also been reported to be expressed by astrocytes and increasingly so after brain injury (Vizuete et al., 1999; Saadoun and Papadopoulos, 2010; Domowicz et al., 2018), was also upregulated in the same areas as that of *Gfap*. The granular distribution of *Aqp4* mRNA-expressing cells provides confirmation that astrocytes are being activated on-site in areas of neuronal damage (Figure 7(h) and (j)).

**Figure 8.**
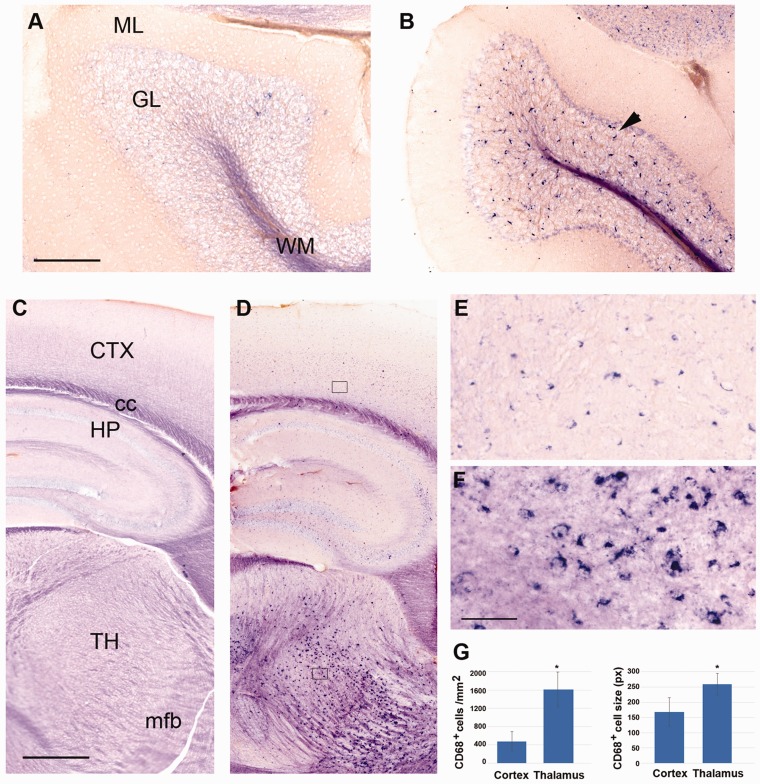
Altered expression patterns of Cd68 in *Tpp1^–^*^/^*^–^* 4-month-old brains. Expression pattern of CD68 mRNA in cerebellum (a and b) and forebrain (c to f) in *Tpp1^–^*^/^*^–^* (b, d, e, f) and control (a, c) 4-month-old brains by *in situ* hybridization (blue staining). (e) and (f) represent an enlargement of sections boxed in (d). Quantification of the number of CD68^+^ cell per mm^2^ and cell size (in pixels) for *Tpp1^–^*^/^*^–^* cortex and thalamus are compared in (g). Black arrowhead indicates CD68^+^ cells in granular cell layer of *Tpp1^–^*^/^*^–^* cerebellum. Scale bars: (a) and (b) = 200 μm; (c) and (d) = 500 μm; (e) and (f) = 50 μm. ML = molecular layer; GL = granular layer; WM = white matter; CTX = cortex; cc = corpus callosum; HP = hippocampus; TH = thalamus; mfb = medial forebrain bundle.

Neuronal damage is effectively sensed by microglia (Ransohoff and Perry, 2009), so it is not surprising that a large number of genes associated with microglia activation were found among the identified DEG (Figure 3). For instance, CD68 is a lysosomal protein that is upregulated in microglia in the brains of several neurodegeneration disorders (Bevan et al., 2018; Linnartz-Gerlach et al., 2019), as well as in human microglia from aged populations (Raj et al., 2017; Olah et al., 2018) and is frequently used as a marker for reactive microglia. Marked elevation of *Cd68* expression was observed in the mRNA quantifications and by ISH in the *Tpp1^–^*^/^*^–^* brains (Figure 8). While CD68-expressing cells are rarely detected in normal brain (Figure 8(a) and (c)), we found elevated *Cd68* expression in morphologically altered microglia throughout the entire 4-month-old *Tpp1^–^*^/^*^–^* brain (Figure 8(b), (d) to (f)). In thalamic and cerebellar areas, these changes were more marked with higher microglia density and clustered expression (Figure 8(b), (d) to (f)), indicating preferential areas of microglia activation. Significant increases in the number and size of CD68^+^ cells were also quantified in thalamus compared with cortex in *Tpp1^–^*^/^*^–^* brains (Figure 8(g)).

### Circadian Rhythm

**Figure 9.**
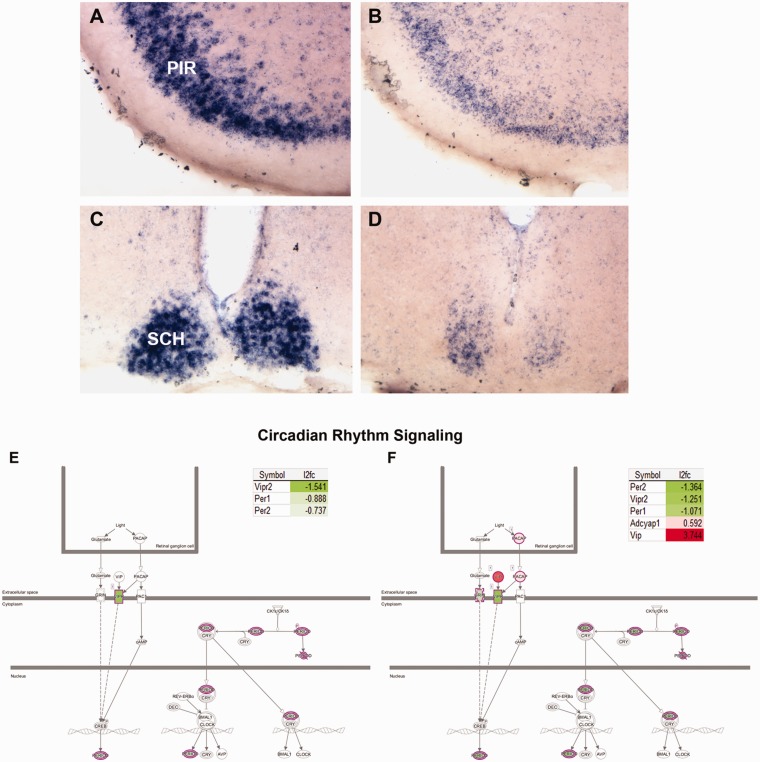
Loss of circadian rhythm gene expression in *Tpp1^–^*^/^*^–^* brains. Loss of Per1 mRNA expression in PIR (a and b) and the SCH (c and d) in *Tpp1^–^*^/^*^–^* (b, d) 4-month-old brains compared with control heterozygous (a, c). Canonical pathway analysis by IPA highlights the genes affected in the circadian rhythm signaling pathways for F/M (e) and Cb (f). Involved DEG detected by RNA-seq analysis and change ratio (l2fc=log_2_ of T vs. N ratio) are indicated. PIR = piriform cortex; SCH = suprachiasmatic nuclei.

Levels of *Per1* and *Per2* were downregulated in the*Tpp1^–^*^/^*^–^* mouse model, which is indicative of circadian rhythm abnormalities and correlates with the altered sleep patterns of Batten patients (Lehwald et al., 2016; Williams et al., 2017). In fact, IPA demonstrated significant alterations in the circadian rhythm pathway (Figure 9); these were further confirmed by the downregulation of *Per1* found by qPCR (Figure 6) and mRNA ISH, which showed neuronal populations of the piriform cortex and the suprachiasmatic nuclei’s being more affected (Figure 9 (a) to (d)). The latter structure is known to have innervations from retinal ganglion cells and is key in coordinating the light cycle with sleep patterns (Abreu and Braganca, 2015; Ono et al., 2015; Roenneberg and Merrow, 2016). Alterations in the circadian rhythm signaling pathways for Cb and F/M are shown in Figure 9 (e and f) and include several genes besides Per1 and Per2. Interestingly, this study includes *Clock* and *Cry* (analyzed by qPCR, see Figure 6(b)) in the list of genes with altered expression in this pathway. In addition, Homer1, one of the rhythmic control transcripts (Maret et al., 2007), was also downregulated in the F/M transcriptome of the *Tpp1^–^*^/^*^–^* mouse brain.

## Discussion

A few reports have analyzed brain gene expression profiles in NCL animal models (*Cln1*, *Cln5*, and *Cln3*; Brooks et al., 2003; Elshatory et al., 2003; von Schantz et al., 2008) as well as in a *cln3^-^ Dictyostelium* model (Mathavarajah et al., 2018). Because most of these reports used microarray methodology and the diseases have different times of onset, data comparison is difficult to perform, and only a few common gene expression changes can be confirmed in *Tpp1^–^*^/^*^–^* brains. Here, we report the first RNA-seq analysis in a Batten disease animal model. Our findings show that the transcriptional characterization of brain gene expression changes due to the lack of TPP1 during the end stages (at 4 months of age) of the disease not only defined the type of neuroinflammation process occurring but also identified a novel affected pathway (i.e., circadian rhythm), both of which contribute to a better understanding of disease pathophysiology in Batten disease (NCL).

In several NCL forms, studies on the involvement of microglia in the inflammation process have been reported. In the most aggressive form of NCL (infantile NCL due to CLN1 mutations), increased proinflammatory cytokine production and peripheral immune cell infiltration of the brain were described (Macauley et al., 2012). Furthermore, anti-inflammatory treatments were successful in increasing the survival rate (Macauley et al., 2012). In animal models of JNCL, characterized by mutations of CLN3, primary microglia were found to be in a proinflammatory state (Xiong and Kielian, 2013), and microglial and astrocytic activation were shown to precede neural cell death (Pontikis et al., 2004). Our study extends these observations to a mouse model of cLINCL in which upregulation of *Cd68*, visualized by ISH (Figure 8), and transcriptional changes associated with microglia (Figure 3 and Supplemental Table S1) confirm its activation through upregulation of cell surface receptors such as *Csf1r*, *Cx3cr1*, *Trem2*, *Tyrobp*, *Tgfr1*, and *Tnfrsf1a*. Similar to AD and in aging (Zhang et al., 2013; Soreq et al., 2017; Mostafavi et al., 2018), microglia constitute the predominant transcriptional footprint in *Tpp1* mutant mouse brain. We also confirmed differential increases in the number of cells in particular brain areas as well as morphological changes (Figure 8) in the *Tpp1^–^*^/^*^–^* brain. Recently, a study using single-cell RNA-seq technology in Tg-AD models revealed a two-step pathway of DAM development; an initial TREM2-independent pathway (Stage 1) followed by a TREM2-dependent pathway (Stage 2). Signatures for both types of microglia stages were found in our analysis of *Tpp1^–^*^/^*^–^* brain (Stage 1 DAM: *Tyrobp*, *Ctsd*, *Apoe*, *B2m*, and *Lyz2*; and Stage 2: *Axl*, *Trem2*, *Itgax*, *Csf1*, *Cst7*, *Lpl*, *Cd9*, *Ccl6*, *Lilrb4a*, and *Timp2*; Keren-Shaul et al., 2017). Thus, it can be expected that microglia in both stages are present in the *Tpp1^–^*^/^*^–^* brain during the end stage of the disease. It remains to be studied whether the Stage-2 DAM that have increased phagocytic activities are functioning normally in the mutant brain, as lysosomal processes might be altered in the absence of TPP1 and defects in microglial function might contribute to the rate of progression of the disease.

It is expected that microglia are initially activated by endogenous ligands generated as a result of neuronal damage, perhaps through toll-like receptors (TLRs; Fiebich et al., 2018) and the complement pathway. Upregulation of genes involved in the complement pathway (Figure 4) is probably linked to microglial and astrocytic response to neuronal damage. Genes from the classical (*C4b*, *C1a*, *C1b*, and *C1c*) and alternate pathways (*C3*) of complement activation as well as its receptor (*C3ar1*) are upregulated. It is possible that activated astrocytes increase the production and release of C3, which in turn interacts with neuronal and microglial C3aR, altering neuronal function and regulation of phagocytosis, respectively, as has been described for models of AD (Lian et al., 2015; Lian et al., 2016; Litvinchuk et al., 2018). TLRs have been shown to have an impact on noninfectious CNS disease and injuries because they can bind a number of endogenous molecules liberated from damaged tissues (Hanke and Kielian, 2011; Fiebich et al., 2018). Involvement of toll receptor signaling was also observed in *Tpp1^–/–^* brains; in particular, *Tlr2* and *Tlr7* were activated in both areas analyzed. In contrast with observations in AD, Parkinson’s disease, and amyotrophic lateral sclerosis patients (Letiembre et al., 2009; Casula et al., 2011), *Tlr4* mRNA was not upregulated in *Tpp1^–/–^* brains, which argues for a unique pathway of microglia activation in this model. It is also important to point out that the three *Tlrs*—*Tlr3*, *Tlr7*, and *Tlr9*—known to be located in endosomes and lysosomes are elevated in Cb (Figure 5 inset) of *Tpp1^–/–^* animals.

Interestingly, two members of the CLN family are upregulated in the *Tpp1^–/–^* brain, cathepsin D (Ctsd), and granulin (Grn), but because these genes have been also found to be upregulated in microglia from other neurodegenerative diseases (i.e., amyotrophic lateral sclerosis), it is unclear if their upregulation in this cLINCL model is related to microglia activation or dysregulation of lysosomal function. Regarding the storage material that characterizes cLINCL, it is interesting that although the subunit C of mitochondrial ATP synthase is the major component of the storage material (Jalanko and Braulke, 2009), no transcriptional changes in the levels of this gene family (*Atp5g1*) were detected, confirming that the accumulation must be due to failure in catabolic protein processing. In contrast, changes related to metabolism of lipids and their transport were extensively represented in the enrichGO analysis (Table S4) in agreement with the multiple lipid alterations described previously for this family of diseases (Kollmann et al., 2013; Kim et al., 2017).

Extensive and severe loss of neurons has been described in cLINCL patients and the *Tpp1*-targeted mouse, and we identified the expression of a large number of neuronal genes affected, particularly in the Cb. Furthermore, the disease and functional networks found by IPA identified genes involved in inflammation of the CNS (Cb, 70 genes; F/M 41 genes). Seizure disorders (Cb, 46 genes), encephalitis (Cb, 65; F/M, 38), and EAE (Cb, 59; F/M, 34) are all known to be correlated with neuronal dysfunction (Table S5). Therefore, it is interesting that the *Tpp1*-targeted neuroinflammation process correlates more strongly with autoimmune types of neurodegenerative diseases (EAE and multiple sclerosis; see Figure 4) than with AD or Parkinson’s disease (Dendrou et al., 2016).

The entry of circulating immune cells into the brain has been described for several neurodegenerative diseases (Minagar et al., 2012; Dendrou et al., 2016; Fiebich et al., 2018), and our data suggest that this process is also active in *Tpp1^–/–^* brains as indicated by the activation of leukocyte extravasation signaling (Figure 4). Furthermore, a large number of endothelium-related changes are part of the set of DEG, suggesting disruption of the blood–brain barrier (Figure 3). Other genetic NCL models also showed compromised blood–brain barrier integrity (Okada et al., 2015; Costa-Silva et al., 2017). The involvement of the Th1 and Th2 activation pathways and Cd28 signaling in T-helper cells, in our model, may indicate T-cell extravasation, but confirmation of this possibility will require further experimental analysis. These pathways in EAE and MS usually involve a compromised blood–brain barrier (Kamimura et al., 2013; Dendrou et al., 2016; Lopes Pinheiro et al., 2016). Further studies will require confirmation of T-cell or other leukocyte infiltration of the brain and, more importantly, determining at which point in time this process is initiated.

Activation of the production of NO and ROS is another event that can lead to pathogenesis during chronic inflammation (Osorio et al., 2007; Urrutia et al., 2014; Gonzalez-Reyes et al., 2017; Bisht et al., 2018). Upregulation of neutrophil factors (*Ncf*) 1, 2, and 4, and cytochrome beta-245 alpha and beta chain genes (*Cybb* and *Cyba*) are indicative of the generation of ROS in *Tpp1^–/–^* brains. Thus, it is intriguing that even though inflammation in cLINCL may be triggered by accumulation of peptides or lipopeptides, similar to that observed in primary neurodegenerative diseases, it may lead to more intricate inflammatory processes that are accelerated by leukocyte infiltration, generation of NO and ROS, and activation of multiple interleukin receptors (listed here), all of which could add to the vicious cycle of neurotoxicity and neuronal death.

Interestingly, two population of reactive astrocytes have been identified, A1 and A2 (Zamanian et al., 2012; Liddelow et al., 2017). A1 upregulates classical components of complement cascade genes and was postulated to be harmful to synapses; A2 upregulates neurotrophic factors and is postulated to be protective (Zamanian et al., 2012). Signatures for both types of reactive astrocytes were found to be upregulated in our transcriptional analysis of *Tpp1^–^*^/^*^–^* brains at 4 months old (A1 type: *Ggta1*, *Gbp2*, *Fbln5*, *Psmb8*; A2 type: *Tgm1*, *Ptx3*, *S100a10*, *Cd109*, *Ptgs2*, *Cd14*). With regard to cross talk between astrocyte and microglia, it has been reported that the release of complement protein C3 activates expression of its receptor C3aR in microglia (Lian et al., 2016). Upregulation of mRNAs for both C3 and its receptor in the *Tpp1^–^*^/^*^–^* brain suggest that peptides and lipids accumulating during disease progression lead to microgliosis via the C3-C3aR pathway that usually engulfs particles through complement-mediated opsonization (Bodea et al., 2014). Conversely, during normal aging microglia may regulate the activation of astrocytes by upregulation of interleukin-1a and complement *Cq1* (Clarke et al., 2018). Both of these components are also found to be upregulated in the *Tpp1^–^*^/^*^–^* brain and thus are possibly involved in sustaining astrocyte activation.

As in other neuroinflammatory diseases (Akiyama et al., 1994; Lue et al., 2001; Gowing et al., 2009), increases in *Csf1* and *Csf1R* expression were also observed in the *Tpp1^–^*^/^*^–^* Cb. It has been suggested that the astrocytic CSF1 interacts with the microglial CSF1 receptor to promote microglial proliferation after injury (Tang et al., 2018), and the coincident localization of activated astrocytes and increased microglial number (Figure 7 and 8) in the *Tpp1^–^*^/^*^–^* brain support this notion. A common feature of neurodegenerative diseases is the presence of activated microglia in areas of neuronal death (Frank-Cannon et al., 2009), while in this cLINCL model, they appear to be in areas of axonal tracts (Figure 8), pointing to the development of an axonopathy as the disease progresses that should be a focus of future research.

Similar to several other neurodegenerative diseases (Heneka et al., 2018), evidence of inflammasome activation through *Nlpr3* expression was detected in the *Tpp1^–^*^/^*^–^* mouse brain, including transcriptional upregulation of key components of that multimolecular signaling complex (i.e., *Aim2*, *Pycard*, *Naip* members, and *Panx1*). This complex serves as a platform to activate inflammatory caspases and the production of interleukin-1, as well as to recognize a broad range of aggregated substances, including perhaps the ones accumulated by lack of TPP1 (lipofuscins).

Furthermore, the dysregulation of phagosome formation and maturation (Table 1) may suggest functional implications related to protein clearance defects associated with the lack of *Tpp1*. Accumulation of phagosomes accompanying disturbed autophagy has been described in photoreceptors on a *Cln3* null mouse model (Cao et al., 2006; Wavre-Shapton et al., 2015). Defective autophagy was also described in several other NCL mouse models (Seranova et al., 2017) as well as in fibroblasts from cLINCL patients (Vidal-Donet et al., 2013); thus, perhaps it is not surprising to find that autophagy pathways are also deregulated in *Tpp1^–/–^* brains. In this model, the implications of increased expression of *Atg9b*, an important component of the early autophagosome structures, will require further scrutiny.

A previous study on the pathological characteristics of the *Tpp1*-targeted mouse remarks that although retinal degeneration is a prominent feature in human cLINCL, in this model, no retinal pathology was observed, and only ascending visual pathways were mildly affected while extensive neurodegeneration was observed in the mouse auditory pathway (Sleat et al., 2004). Thus, our findings that there are global circadian rhythm abnormalities in this animal model may indicate that these effects are not necessarily linked to visual disturbances but rather to a more generalized effect, as we confirmed downregulation of *Per1*, not only in the suprachiasmatic nuclei but also in the piriform cortex (Figure 9) and Cb. In the Cb, clock gene rhythms have been detected, and the Cb circadian oscillators have been implicated in the response to food anticipation (Mendoza et al., 2010). Thus, it is feasible to suggest that the sleep abnormalities and food-intake disturbance seen in Batten’s patients may be linked to cerebral oscillator abnormalities. Furthermore, our findings in this model offer a molecular basis to investigate patients’ sleep disorders symptoms, which open a novel area of research for this disease.

In summary, this study highlights the prominent involvement of neuroinflammation and oxidative stress in the development of NCL due to lack of TPP1 activity. The initial lysosomal pathway defect associated with a lack of TPP1 leads to generation of lipid/protein aggregates and axonal toxicity that triggers the neuroinflammation process. This process is potentiated by astrocyte–microglia communication, leading to oxidative stress and, probably, disruption of the blood–brain barrier. Several neuroinflammation aspects were found to be unique to this model when compared with other neurodegenerative diseases, and this information should help tailor appropriate therapeutics for NCLs in the future. Considering the extensive involvement of microglia, astrocytes, and endothelial cells in the pathology, neuroinflammation, and neurodegenerative process, and that they are themselves compromised in their function due to the lack of TPP1, it is important to establish their potential as therapeutic targets for this disease. It will be also important to extend these findings and provide an *in vivo* timeline for understanding the developmental progression of the disease, in particular as it relates to the progression of the neuroinflammation state and the identification of initial triggers and responders to the dysfunctional lysosomal paradigm.

## Summary

Brain transcriptome analysis in the *Tpp1^–/–^* mouse line identified specific brain inflammation pathways in which microglia and astrocyte activation potentiates neuronal dysfunction possibly by oxidative stress and damage of the blood–brain barrier integrity. Defects on the control of circadian rhythm were also observed.

## Supplementary Material

Supplemental Table S1

Supplemental Table S2

Supplemental Table S3

Supplemental Table S4

Supplemental Table S5
